# Programmed death ligand 1-positive immune cells in primary tumor or metastatic axillary lymph nodes can predict prognosis of triple-negative breast cancer even when present at < 1% in the tumor region

**DOI:** 10.1007/s12282-023-01442-9

**Published:** 2023-03-09

**Authors:** Nobumoto Tomioka, Kanako C. Hatanaka, Dai Okuyama, Ken-ichi Watanabe, Mitsugu Yamamoto, Hideki Maeda, Hanae Tachikawa, Sayuri Kuwahara, Ai Shimizu, Hiroaki Suzuki, Yutaka Hatanaka, Masato Takahashi

**Affiliations:** 1 Department of Breast Surgery, National Hospital Organization (NHO) Hokkaido Cancer Center, 4-2 Kikusui, Shiroishi-ku, Sapporo, 003-0804 Japan; 2 Clinical Research and Medical Innovation Center of Development of Advanced Diagnostics, Hokkaido University Hospital, Sapporo, Japan; 3 Department of Clinical Pathology, National Hospital Organization (NHO) Hokkaido Cancer Center, Sapporo, Japan; 4 Department of Surgical Pathology, Hokkaido University Hospital, Sapporo, Japan; 5 Research Division of Genome Companion Diagnostics, Hokkaido University Hospital, Sapporo, Japan; 6 Department of Breast Surgery, Hokkaido University Hospital, Sapporo, Japan

**Keywords:** PD-L1, TILs, TNBC, TME, ICI

## Abstract

**Background:**

The efficacy of pre-operative systemic treatment (PST) combined with immune checkpoint inhibition (ICI) for triple-negative breast cancer (TNBC) has been recognized recently as being independent of the degree of programmed death ligand-1 (PD-L1) positivity of infiltrating immune cells, especially for patients with axillary lymph node metastasis (ALNM).

**Methods:**

TNBC patients with ALNM were treated surgically between 2002 and 2016 in our facility (n = 109), of whom 38 received PST before resection. The presence of tumor-infiltrating lymphocytes (TILs) expressing CD3, CD8, CD68, PD-L1 (detected by antibody SP142) and FOXP3 at primary and metastatic LN sites was quantified.

**Results:**

The size of invasive tumor and the number of metastatic axillary LN were confirmed as prognostic markers. The numbers of both CD8+ and FOXP3+ TILs at primary sites were also recognized as prognostic markers, especially for overall survival (OS) (CD8, p = 0.026; FOXP3, p < 0.001). The presence of CD8+, FOXP3+ and PD-L1+ cells was better maintained in LN after PST and may contribute to improved antitumor immunity. Provided they were present as clusters of ≥ 70 positive cells, even < 1% of immune cells expressing PD-L1 at primary sites predicted a more favorable prognosis for both disease-free survival (DFS) (p = 0.004) and OS (p = 0.020). This was the case not only for 30 matched surgical patients, but also in all 71 surgical only patients (DFS: p < 0.001 and OS: p = 0.002).

**Conclusions:**

PD-L1+ , CD8+ or FOXP3+ immune cells in the tumor microenvironment (TME) at both primary and metastatic sites are significant on prognosis, which could be a clue to expect the potential for better responses to the combination of chemotherapy and ICI, especially for patients with ALNM.

## Introduction

Refractory disease is unfortunately sometimes the prognosis for TNBC despite the administration of chemotherapy recommended as PST. Recently, encouraging results have emerged from clinical trials combining ICI using agents such as atezolizumab or pembrolizumab with standard chemotherapy as PST regardless of the PD-L1-positivity in the intend-to-treat (ITT) population [1, 2]. Patients with ALNM were better responders by subgroup analysis in each of these trials. It is therefore important to investigate associations between tumor cells and different immune cells infiltrating not only the primary site but also the regional metastatic LNs in patients with or without PST. This can be accomplished by evaluating immune cell markers such as CD3, CD8, PD-L1, FOXP3 and CD68, in order to acquire a comprehensive understanding of the TME from temporal, spatial and quantitative viewpoints [3].

## Materials and methods

### Patients and samples

Of the total of 3902 patients who underwent surgery between January 2002 and December 2016 at our facility, 503 had estrogen receptor (ER)-negative and her2/neu (HER2)-negative breast cancer, and of these, 109 TNBC patients with ALNM were recognized and included in the present study. This was conducted in full accordance with ethical principles including the Helsinki Declaration and was approved by the Institutional Review Board of the Hokkaido Cancer Center. We acquired consent from all patients at the time of admission to use their specimens as well as clinical data. Of the 109 TNBC patients, 71 received surgery followed by adjuvant chemotherapy (henceforth referred to as surgery only), and 38 received PST before surgery (Table 1). Patients received anthracycline and taxane sequentially (e.g., 3wECx4/FECx4 and 3wDTXx4/wPTXx12) as PST or basic adjuvant chemotherapy. Two of the 38 patients received only wPTXx12 as PST. Two experienced a confirmed pathological complete response (pCR) and another two had almost a pCR of the primary lesion. The axillary metastatic LN of these patients were examined.

**Table 1:** Clinical characteristics of the TNBC patients with ALNM

TNBC	Surgery only	PST administered	*p*
Number of patients	71	38	
Age (mean)	36–78 (57.7)	30–79 (54.7)	
pT (cm)			0.318
< 2	20	15	
2 < ≤ 5	39	17	
5 <	11	6	
pN (nodes)			0.471
1–3	47	20	
4–9	9	12	
10 <	15	6	
Recurrence			0.461
–	51	13	
+	20	24	
Outcome			0.558
Alive	50	12	
Dead	21	26	
(Death from other causes)	1	3	

### Hematoxylin & eosin (H&E) staining, and immunohistochemistry

**Fig. 1:**
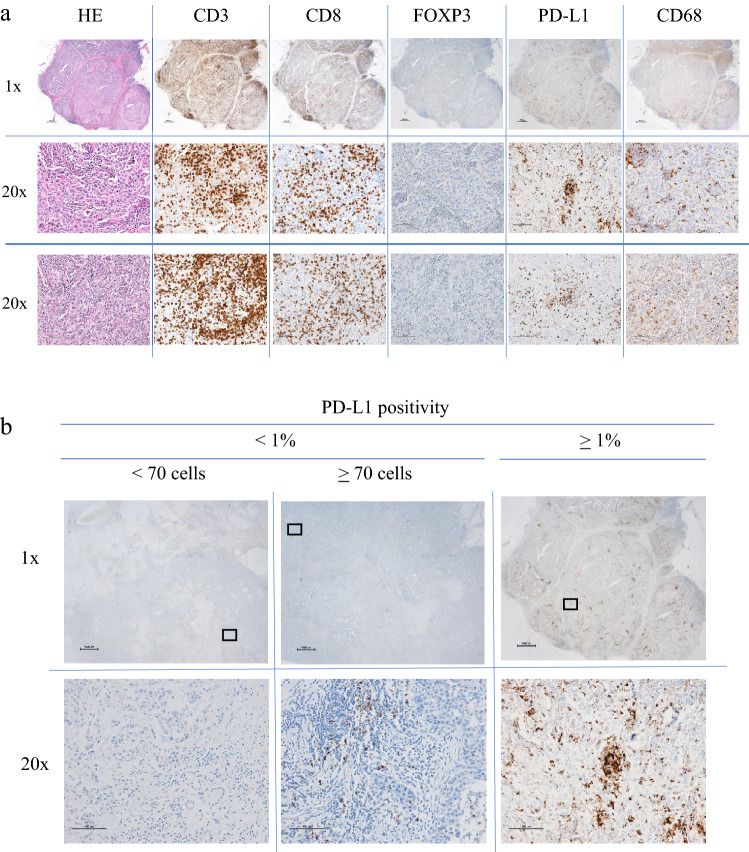
**a** Example of immunohistochemistry of immune cell staining for CD3, CD8, FOXP3, PD-L1, CD68 and H&E staining of the primary tumor and metastatic LN. The upper two rows are primary sites (1×, 20×), and the bottom row shows metastatic LN (20x). **b** PD-L1 (SP142) positivity of immune cells in the primary tumor. Left panels: < 1% and < 70 cells; Center panels: < 1% but ≥ 70 cells; Right panels: ≥ 1% and ≥ 70 cells. Upper row is 1× magnification of primary site, bottom row is the same 20x

H&E staining and immunohistochemistry were performed on formalin-fixed paraffin-embedded surgical specimens sectioned at 4 μm thickness and mounted on glass slides. Immunohistochemistry for PD-L1 (clone SP142, dilution 1:20; Roche, Germany) and FOXP3 (clone 236A/E7, dilution 1:200; Abcam, USA) was performed using a BenchMark ULTRA automated stainer (Ventana Medical System Inc., Tucson AZ, USA). For PD-L1, deparaffinization, epitope retrieval, and immunostaining were performed using Cell Conditioning solutions and the OptiVIEW Universal DAB detection system according to the manufacturer’s instructions. Positive signals were amplified using OptiView Copper and sections were counterstained with Mayer’s hematoxylin. Immunohistochemistry for CD3 (clone LN10, Leica, Germany), CD8 (clone 4B11, Leica) and CD68 (clone 514H12, Leica) was performed using a Leica BOND-III automated stainer (Leica Biosystems Inc, Wetzlar Hesse, Germany) with pre-diluted antibodies, Novocastra HD BOND reagents and BOND Compact Polymer Detection solutions employed according to the manufacturer’s instructions. In each run, appendix sections were included as positive controls (Fig. 1a).

### Evaluation of CD3, CD8, PD-L1, FOXP3 and CD68 expression by TILs

We identified the area of the tumor region, then quantified the amount of TILs therein. Next, we quantified the proportions of cells positive for CD3, CD8, CD68, PD-L1 and FOXP3 in each tumor region both in primary and metastatic lesions in axillary LNs. These were categorized as 0%, < 1%, or 1, 2, 3, 4, 5, 6, 7, 8, or 9% and for larger fractions in 10-percentiles (10, 20, 30, 40, 50, 60, 70, 80, 90, 100% rounding up to the nearest 10% value). All estimations were based on pathologists’ evaluations counted by eye under 20 × magnified microscopic views. If there was a significant difference in the value of the evaluation between researcher and pathologist, it was supposed to be re-evaluated after regular conferences.

### Enumerating PD-L1-positive immune cells using the SP142 antibody

**Fig. 2:**
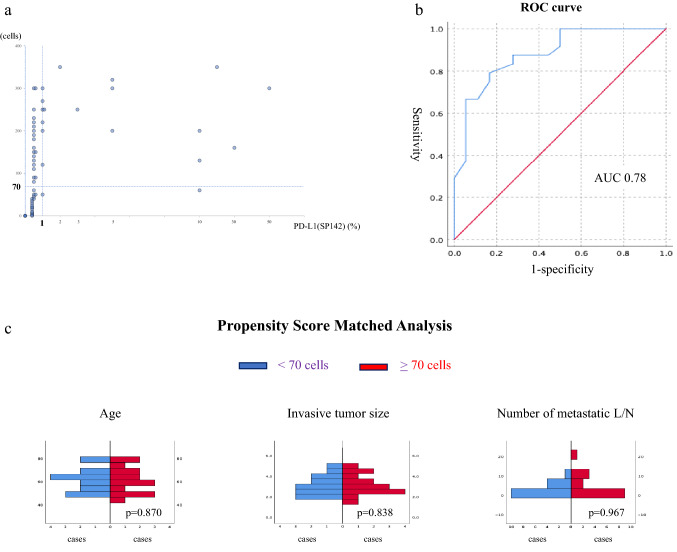

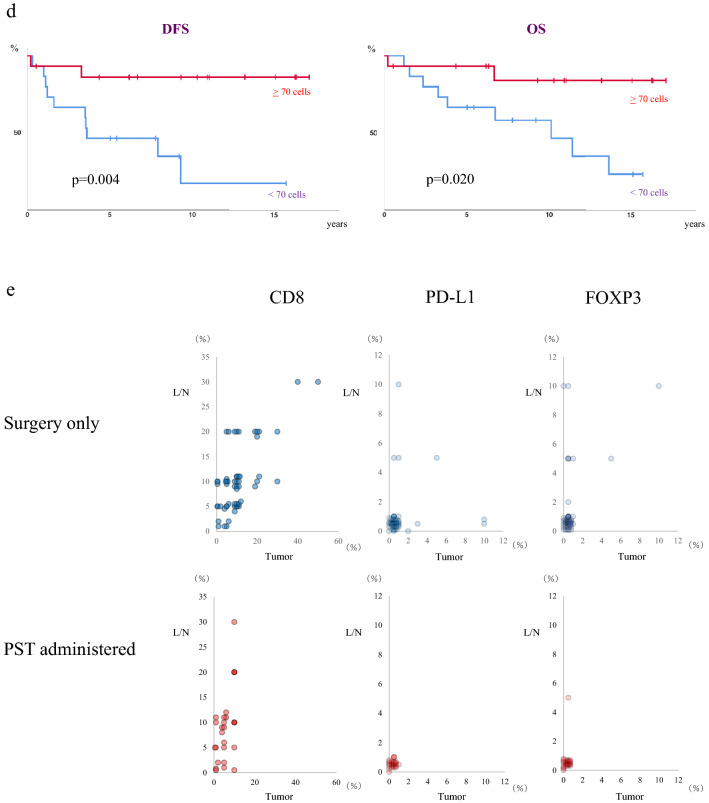
**a** Distributions of the number of PD-L1(SP142)+ immune cells at primary sites for patient receiving surgery only. **b** ROC curve, showing an AUC of 0.78, for survival status with or without recurrence. It was decided to select 70 PD-L1(SP142)+ immune cells as the threshold for the prediction of recurrence. **c** PSM analysis for the 3 factors, age of patients, size of invasive tumors and number of metastatic LNs, with 15 patients selected in each group. **d** DFS and OS of the 30 PSM patients stratified by the threshold of 70 PD-L1(SP142)+ immune cells. **e** Distribution of immune cells positive for CD8, PD-L1(SP142) or FOXP3 at primary sites (tumor) and metastatic axillary lymph nodes (L/N)

A previous study had reported ≥ 1% of cells positive for the PD-L1 antibody SP142 in only around 40% of patients with recurrent TNBC [4]. A similar result was obtained in the present study. On the other hand, PD-L1 (SP142)-positive immune cells were commonly observed at a frequency of < 1%. We counted the number of PD-L1-positive immune cells in each cluster in the area to be evaluated, and took the greatest number among the clusters as the analytical value for each patient (Figs. 1b, 2a). We employed receiver operating characteristic (ROC) curves to estimate appropriate threshold values for exact prediction of the recurrence and evaluate it for the OS for the patients with < 1% PD-L1 positivity (Fig. 2b). Furthermore, to obviate the influence of clinicopathological factors such as age, size of tumor and number of metastatic LNs, propensity score matching (PSM) was also performed for patients with < 1% PD-L1+ cells, in order to increase reliability (Fig. 2c).

### Statistical analyses

Wilcoxon’s signed-rank tests, Mann–Whitney tests, the Kaplan–Meier method, Log-rank test, Cox’s proportional hazard ratios, ROC and PSM analyses were all carried out using IBM SPSS software version 26, taking a p value of < 0.05 as significant.

## Results

### Less than 1% of TILs expressing PD-L1 in the TME predicts the prognosis of TNBC patients

First, we analyzed samples from 71 patients who received surgery without PST followed by adjuvant chemotherapy (“surgery only”), of whom 46 (65%) exhibited TILs with < 1% PD-L1 positivity. The mean absolute number of PD-L1+ cells counted was 85, median 50, maximum 350 (Fig. 2a). ROC curves for the numbers of PD-L1+ immune cells for the event of recurrence exhibited an area-under-the-curve (AUC) of 0.78, and it emerged that the most suitable threshold value was 70 positive cells for the most accurate prediction of recurrence (Fig. 2b), and it worked for DFS and also OS of the 46 patients with significance (Table 2). Furthermore, to obviate the impact of clinicopathological features, PSM was performed for patient age, tumor size and number of metastatic LNs. This resulted in the inclusion of 30 patients of the 46 with < 1% PD-L1+ cells (Fig. 2c). The 15 patients with < 70 PD-L1+ immune cells had worse DFS and OS than the other 15 matched patients who had ≥ 70 although still with < 1% PD-L1+ cells (p = 0.004, p = 0.020) (Fig. 2d, Table 2). This threshold value has been able to distinguish between patients with a less favorable prognosis among all 46 patients with < 1% PD-L1+ cells for both DFS and OS (p < 0.001, p = 0.012, respectively) (Table 2). Furthermore, by including the 25 patients with ≥ 1% PD-L1+ cells in the group of those with ≥ 70 positive cells but still < 1% of total (n = 71 altogether), the discriminatory potential of the 70 cells cutoff was retained or even enhanced (p < 0.001, p = 0.002) (Table 2). On the other hand, evaluating all 71 patients using a threshold of ≥ or < 1% PD-L1+ immune cells, or a threshold of ≥ or < 30% of TILs as evaluated by H&E staining, fails to establish significant differences in neither DFS (p = 0.138 and p = 0.154, respectively), nor OS, except for OS by TILs (p = 0.1111 and p = 0.004) (Table 2).

**Table 2:** DFS and OS of patients stratified according to the cut-off of 70 PD-L1(SP142) + immune cells

70 cells	1%		DFS (*p*)	HR/95% CI	OS (*p*)	HR/95% CI
< 70 cells (15 matched) vs	≥ 70 cells but < 1% (15 matched)		0.004	0.140 (0.030–0.657)	0.020	0.195 (0.042–0.904)
All < 70 cells (26) vs	All ≥ 70 cells but < 1% (20)		< 0.001	0.058 (0.008–0.436)	0.012	0.231 (0.066–0.805)
All < 70 cells (26) vs	All ≥ 70 cells but < 1% (20)	& all ≥ 1% (25)	< 0.001	0.177 (0.073–0.432)	0.002	0.280 (0.117–0.669)
	All < 1% (46) vs	All ≥ 1% (25)	0.138	0.503 (0.199–1.270)	0.154	0.491 (0.181–1.332)
	TILs < 30% (55) vs	TILs ≥ 30% (16)	0.1111	0.506 (0.216–1.189)	0.004	0.308 (0.132–0.719)

### Correlations of CD8 + , FOXP3 + and PD-L1 + immune cell infiltration are differently affected by PST in primary sites and ALNM

Next, correlations of the degree of infiltration of CD8+ or FOXP3+ with PD-L1+ immune cells were evaluated for primary sites and ALNM. For patients receiving only surgery, correlation coefficients for CD8+ or FOXP3+ cells with PD-L1+ immune cells were 0.591 and 0.445, respectively, at primary sites, and 0.688 and 0.546 in ANLM (all p < 0.001) (Table 3a). Thus, moderate correlations were observed both in primary sites and ALNM, but for patients receiving PST, these correlations were different. For CD8+ and PD-L1+ cells they were 0.060 (p = 0.734) and 0.637 (p < 0.001), respectively, at primary sites and in ALNM. For FOXP3+ and PD-L1+ cells, these values were 0.983 (p < 0.001) and 0.237 (p = 0.176), respectively (Table 3b) (Fig. 2e). This analysis demonstrated that the degree of infiltration of PD-L1+ immune cells after administering PST correlated better with CD8 + immune cells in ALNM than at primary sites, but better with FOXP3+ cells at primary sites than in ALNM.

**Table 3:** Correlation coefficients between PD-L1+ and CD8+ or FOXP3+ cells at the primary site or metastatic LNs in patients with or without PST

	Correlation coefficient (*p*)	CD8	FOXP3
Surgery only			
Primary	PD-L1	0.591 (p < 0.001)	0.445 (p < 0.001)
Ax lymph node	PD-L1	0.688 (p < 0.001)	0.546 (p < 0.001)
PST administered			
Primary	PD-L1	0.060 (p > 0.734)	0.983 (p < 0.001)
Ax lymph node	PD-L1	0.637 (p < 0.001)	0.237 (p > 0.176)

### Absolute numbers of both PD-L1 + and CD8 + are maintained in ALNM after PST

Comparing surgery alone with PST patients, and comparing primary lesions with ALNM, we evaluated differences in the degree of infiltration by CD3+ , CD8+ , PD-L1+ , FOXP3+ and CD68+ cells. For PD-L1+ immune cells, this was not different at primary sites and ALNM in patients who received only surgery (p = 0.887). However, these cells were decreased at primary sites in patients receiving PST, relative to those with surgery alone (p < 0.001), but the absolute numbers were maintained in the ALNM (p = 0.100 by Mann–Whitney testing; p = 0.021 by Wilcoxon signed-rank testing) (Table 4a). The patterns of infiltrating CD8 + cells were quite similar (p = 0.892, p < 0.001, p = 0.335, p = 0.016) (Table 4b).

**Table 4:** CD8+ , PD-L1+ , FOXP3+ , and CD68+ immune cells at the primary site or metastatic LN of patients with or without PST

	Surgery only	PST administered	Mann–Whitney test (*p*)
(a) PD-L1			
Primary (%)	3.2 ± 7.8%	0.5 ± 0.8%	< 0.001
Lymph nodes (%)	3.1 ± 6.7%	1.4 ± 3.7%	0.100
Wilcoxon signed-rank test (*p*)	0.887	0.021	
(b) CD8			
Primary (%)	10.9 ± 7.2%	6.0 ± 4.1%	< 0.001
Lymph nodes (%)	11.0 ± 9.1%	9.6 ± 9.0%	0.338
Wilcoxon signed-rank test (*p*)	0.892	0.016	
(c) FOXP3			
Primary (%)	0.7 ± 1.4%	0.5 ± 0.8%	0.061
Lymph nodes (%)	1.6 ± 2.5%	0.8 ± 1.1%	0.030
Wilcoxon signed-rank test (*p*)	< 0.001	0.018	
(d) CD68			
Primary (%)	14.7 ± 11.3%	7.4 ± 4.2%	< 0.001
Lymph nodes (%)	11.9 ± 9.0%	10.7 ± 6.9%	0.818
Wilcoxon signed-rank test (*p*)	0.031	0.093	

### The greater infiltration of FOXP3 + immune cells into ALNM than primary sites is maintained even after PST

Infiltration by FOXP3+ immune cells into ALNM was greater than at primary sites in patients receiving surgery alone (p < 0.001), but decreased more in ALNM for patients given PST (p = 0.030). However, absolute numbers of FOXP3 + cells remained higher in the ALNM than at primary sites after PST (p = 0.018) (Table 4c). For CD68+ immune cells, there were more at the primary sites than ALNM (p = 0.031), decreasing more at primary sites after PST (p < 0.001) but maintained in the ALNM even after PST (p = 0.818) (Table 4d).

### Robust and reliable prognostic factors identified by proportional hazards analyses

We compared the proportional hazard ratios for DFS and OS in patients receiving only surgery with those also receiving PST, using a forward stepwise approach with 6 factors, as follows: size of invasive tumor, number of metastatic lymph nodes, and the degree of infiltration of CD8+ , PD-L1+ , FOXP3+ or CD68+ immune cells at primary site as a percentage of absolute values. The prognostic factors identified for DFS of patients receiving surgery alone were size of invasive tumor (HR 1.290, p < 0.007) and FOXP3+ infiltration (HR 1.461, p < 0.005) (Table 5a), but for patients receiving PST, they were size of invasive tumor (HR 1.219, p < 0.003) and number of metastatic lymph nodes (HR 1.115, p < 0.032) (Table 5b). Prognostic factors for OS after surgery alone were the degree of infiltration of CD8 + (HR 0.904, p < 0.026) and FOXP3+ immune cells (HR 1.733, p < 0.001) (Table 5c), but for the patients with PST, these were the size of invasive tumor (HR 1.247, p < 0.001) and number of metastatic lymph nodes (HR 1.117, p < 0.033) (Table 5d). Thus, for patients receiving PST, tumor size and the number of metastatic LNs are consistent prognostic factors for both DFS and OS. On the other hand, for patients receiving surgery only, CD8+ and FOXP3+ cell infiltration plays a greater role for predicting prognosis. These findings suggest that the status of these immune cells might have some impact on established clinicopathological prognostic factors, such as size of invasive tumor or number of metastatic lymph nodes, as seen after administering PST.

**Table 5:** Prognostic factors for DFS/OS for patients with or without PST

	*p*	HR	95% CI
(a) DFS for surgery only			
Invasive tumor size	0.007	1.290	1.073–1.551
FOXP3	0.005	1.461	1.125–1.898
(b) DFS for PST administered			
Invasive tumor size	0.003	1.219	1.070–1.388
Number of metastatic LN	0.032	1.115	1.009–1.231
(c) OS for Surgery only			
CD8	0.026	0.904	0.827–0.988
FOXP3	< 0.001	1.733	1.254–2.393
(d) OS for PST administered			
Invasive tumor size	< 0.001	1.247	1.091–1.424
Number of metastatic LN	0.033	1.117	1.009–1.237

## Discussion

Comparing the site-specific TME between primary sites and ALNM, and differences resulting from administering PST or not, revealed informative differences in the degree of infiltration of various immune cells in this study. First, we focused on the status of PD-L1+ immune cells detected by the antibody SP142 at primary sites in patients receiving only surgery. Some clusters of these cells comprising < 1% of all cells in the tumor region were seen in 46 of the 71 patients in this study (65%), which is similar to the around 60% in a previous report of the IMpassion 130 study [4]. Because we hypothesized that even < 1% positivity for PD-L1 might still have clinical implications, we enumerated these and applied ROC and PSM analyses. We found that a threshold of an absolute number of 70 PD-L1+ immune cells was potentially a predictive marker for the efficacy of adjuvant chemotherapy and patient prognosis. We acknowledge that this value might have been influenced by the small number of patients and the limitation of this study is not to have validation set for that value, but still it seems reliable as confirmed through the PSM analysis considering age and the two established prognostic factors, the size of invasive tumor and the number of metastatic lymph nodes. Despite the paucity of PD-L1+ immune cells when < 1% in the tumor region, our findings suggest that this amount is sufficient to predict the response in primary lesions or metastatic lymph nodes. Thus, the number of PD-L1+ cells was a quantitative favorable prognostic marker for TNBC with ALNM even when the fraction of PD-L1+ cells was < 1% in the tumor region. Such PD-L1+ cells were observed to be present in clusters, possibly representing tertiary lymphoid structures (TLS) at different stages of maturity [5]. We are currently further evaluating these clusters for CD20, CD21 and MECA79 expression in this sample set. Preliminary data indicate that the numbers of PD-L1+ cells correlate more closely with CD20+ cells than CD8+ cells, and the numbers of CD21+ cells correlate with MECA79+ cells. If small clusters of PD-L1+ cells were related to a very early stage of the TLS formation process, which could have a significant impact on prognosis, this would be consistent with the prognostic potential of PD-L1+ clusters and would explain the therapeutic effect of ICI regardless of PD-L1 positivity in the intend-to-treat (ITT) population in clinical trials such as IMpassion 031 or KEYNOTE 522 [1, 2].

The number of CD8+ immune cells correlated with PD-L1+ cells as well as with CD3+ cells (data not shown) at the primary sites for patients receiving only surgery. Dense infiltration of PD-L1+ immune cells was not simply due to the presence of greater numbers of TILs. This emphasizes a possible enrichment of T cells with potential immunological specificity for the tumor, consistent with the status of PD-L1+ immune cell infiltration as a predictor of prognosis for TNBC with ALNM. Furthermore, the analyses of these paired samples suggested that the TME is better maintained in axillary LNs than at the primary site, as indicated by the influence of PST especially on infiltration of CD8+ and PD-L1+ immune cells. This could indicate that TNBC patients with ALNM would benefit from ICI, potentially because of enhanced priming in LNs followed by increased effector cells at the primary sites or occult metastatic sites. Additionally, FOXP3+ cells present in the axillary LNs were decreased after PST, albeit more were still present than at the primary sites. Hence they could be candidate therapeutic targets, especially because TNBC tends to recur in regional LNs, which could provide a rationale for axillary LN dissection at this time.

We compared prognostic factors for DFS between patients with or without PST, and found that the degree of infiltration of FOXP3+ cells seemed to be related to the number of metastatic LNs, reflecting the efficacy of PST. Although speculative, in a similar manner, the influence of the degree of infiltration of CD8+ immune cells on OS could also be related to the size of invasive tumor again reflecting the efficacy of PST. Presumably, the degree of infiltration of CD8+ or FOXP3+ immune cells at primary sites might predict the efficacy of adjuvant chemotherapy not only for primary sites and regional LNs but even for subclinical micrometastases at other organ sites, and thereby influence DFS and OS benefits.

In spite of the importance of therapeutic approaches directed to FOXP3+ immune cells, many of which are regulatory T cells (Tregs), administration of both anti-CTLA-4 and anti-PD1 antibodies might nonetheless result in hyper progressive disease (HPD) because both CTLA-4 and PD-1 are expressed by Tregs [6, 7]. Moreover, immune cells expressing PD-L1 would not suppress Tregs through cis-PD-L1/CD80 interactions [8]. On the other hand, PD-L1 positivity of tumor cells results in different effects to those mediated by PD-L1 expressed by immune cells. Intrinsically upregulated PD-L1 on tumor cells could increase their malignant potential by translocating from the plasma membrane to their nuclei and result in expression of genes modulating anti-tumor immune responses [9]. Furthermore, tumor cells do not themselves trigger cis-PD-L1/CD80 interactions which result in survival of tumor cells through PD-1/PD-L1 interactions [8]. Patients with an unfavorable prognosis due to PD-L1+ tumor cells and fewer TILs [10], especially with less infiltration of PD-L1+ immune cells, would be the next candidates for therapeutic targeting to prevent tumors escaping immune surveillance. In light of these considerations, PD-L1 positivity as assessed using the 22C3 antibody would be preferred for predicting the efficacy of ICI treatment, even though the SP142 antibody seems to predict prognosis based on specific TILs [11]. Recently, the TME status of the liver has also been elucidated [12, 13], and less PD-L1+ immune cells infiltrating around the metastatic sites has been reported [4].

In conclusion, this study might infer the existence of a population in which an immune response could be expected even when PD-L1 was less than 1%, which may be one of the reasons for the additive effect of ICI in PST, regardless of PD-L1 positivity. In order to determine a therapeutic approach to improve the prognosis of refractory TNBC in the future, it is expected to investigate specific TME not only in the LN but also in each organ, regardless of whether metastasis is clinically observed or not, and to devise ways to enhance tumor immunity in those sites.

## Data Availability

We would like to perform disclosure and the discussion of data with pleasure anytime.
