# Correction: Programmed death ligand 1-positive immune cells in primary tumor or metastatic axillary lymph nodes can predict prognosis of triple-negative breast cancer even when present at < 1% in the tumor region

**DOI:** 10.1007/s12282-023-01454-5

**Published:** 2023-04-27

**Authors:** Nobumoto Tomioka, Kanako C. Hatanaka, Dai Okuyama, Ken-ichi Watanabe, Mitsugu Yamamoto, Hideki Maeda, Hanae Tachikawa, Sayuri Kuwahara, Ai Shimizu, Hiroaki Suzuki, Yutaka Hatanaka, Masato Takahashi

**Affiliations:** 1 Department of Breast Surgery, National Hospital Organization (NHO) Hokkaido Cancer Center, 4-2 Kikusui, Shiroishi-ku, Sapporo, 003-0804 Japan; 2 Clinical Research and Medical Innovation Center of Development of Advanced Diagnostics, Hokkaido University Hospital, Sapporo, Japan; 3 Department of Clinical Pathology, National Hospital Organization (NHO) Hokkaido Cancer Center, Sapporo, Japan; 4 Department of Surgical Pathology, Hokkaido University Hospital, Sapporo, Japan; 5 Research Division of Genome Companion Diagnostics, Hokkaido University Hospital, Sapporo, Japan; 6 Department of Breast Surgery, Hokkaido University Hospital, Sapporo, Japan

**Correction: Breast Cancer** 10.1007/s12282-023-01442-9

In the original publication of the article, in table 2, the second column heading “1%” should be aligned to the value “& all>1%(25)” in third row. The correct Table 2 is given in this correction.

**Table 2:**
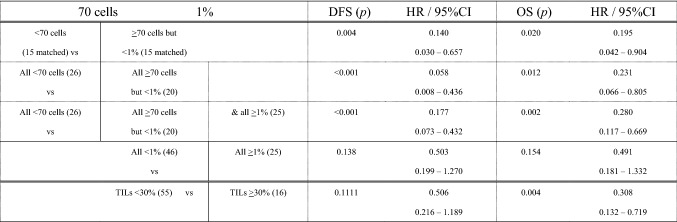
DFS and OS of patients stratified according to the cut-off of 70 PD-L1(SP142)+ immune cells

